# The transmembrane domains mediate oligomerization of the human ZIP4 transporter in vivo

**DOI:** 10.1038/s41598-022-24782-6

**Published:** 2022-12-06

**Authors:** Yuting Liu, Elizabeth M. Bafaro, Ann E. Cowan, Robert E. Dempski

**Affiliations:** 1 Department of Chemistry and Biochemistry, Worcester Polytechnic Institute, 100 Institute Road, Worcester, MA 01609 USA; 2 Department of Molecular Biology and Biophysics and Center for Cell Analysis and Modeling, University of Connecticut, Farmington, CT 06030 USA

**Keywords:** Permeation and transport, Ion transport, Metalloproteins

## Abstract

The human (h) ZIP4 is a plasma membrane transporter that functions to increase cytosolic zinc levels. hZIP4 encodes eight transmembrane domains and a large extracellular domain (ECD). This ECD is cleaved from the holo-transporter when cells are zinc-deficient. At the same time, mutations in the ECD can result in the zinc-deficiency disease *Acrodermatitis enteropathica*. Previously, it was shown that hZIP4’s ECD is comprised of two structurally independent subdomains where contacts between the ECD monomeric units are centered at the PAL motif. These results lead to the hypothesis that ZIP4-ECD is essential to the dimerization of the holo-transporter. To test this hypothesis, we used Fluorescence Correlation Spectroscopy (FCS) to quantify the oligomeric state of full-length hZIP4 and hZIP4 lacking the ECD domain, each tagged with eGFP. Inspection of our experimental results demonstrate that both the full-length and truncated hZIP4 is a dimer when expressed in HEK293 cells. Parallel functional experiments demonstrate that the *K*_*m*_ and *V*_*max*_ for truncated and full-length hZIP4/eGFP are similar. Determining that truncated hZIP4/eGFP forms a dimer is a crucial step for understanding the function of the hZIP4-ECD, which provides more insight into how the diseases related to hZIP4 protein.

## Introduction

Zn^2+^ is an essential trace element involved in various biological functions^1^. Up to 10% of human proteins associate with Zn^2+^ as Zn^2+^ provides structural stability for hundreds of enzymes^2^. For instance, Zn^2+^ stabilizes the fold of zinc finger proteins^3^. There is also increasing evidence that Zn^2+^ functions as a signaling molecule^4^.

**Figure 1:**
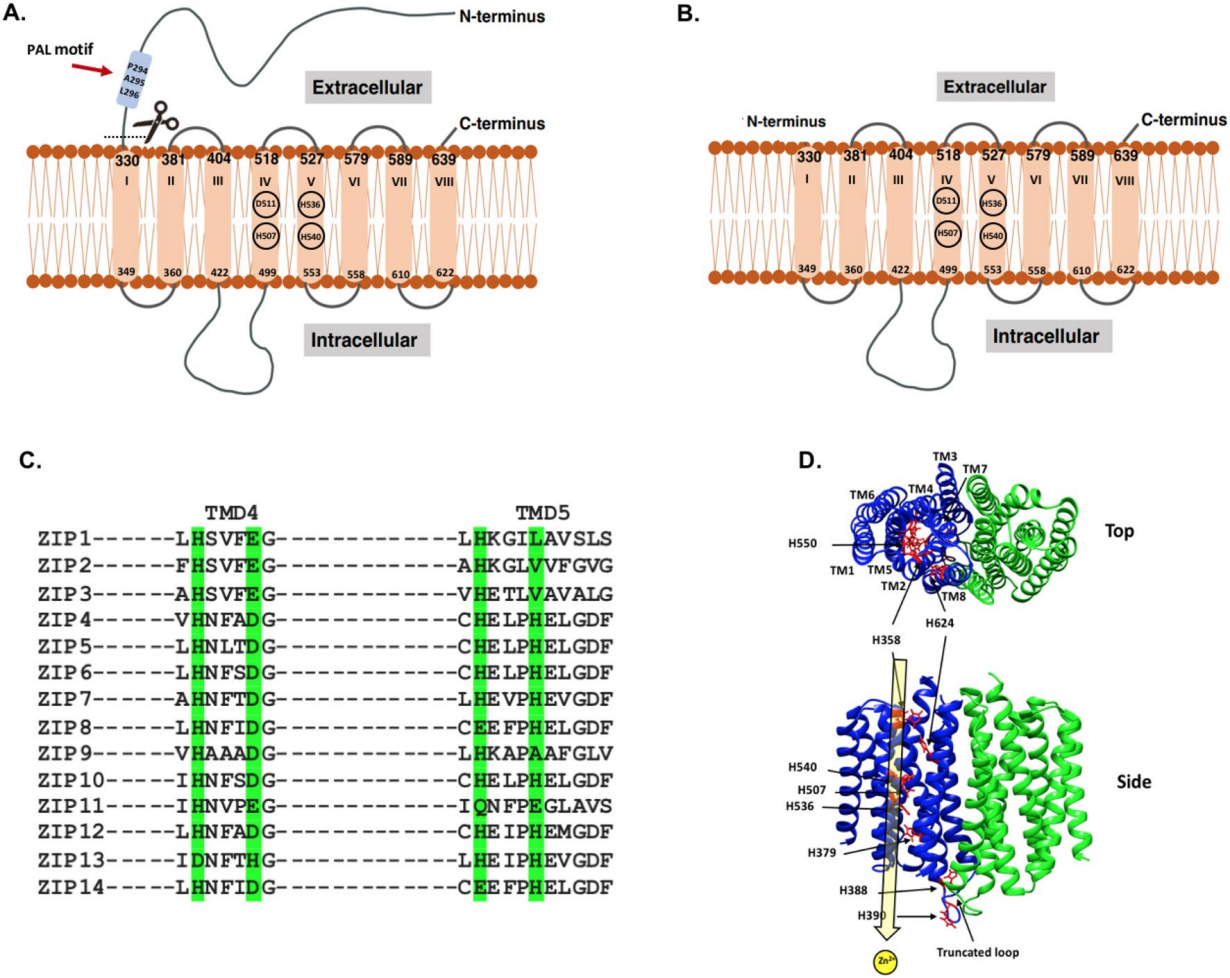
The topology of ZIP4 (**A**) and truncated ZIP4 (**B**), the sequence alignment of ZIP transmembrane domain (TMD) 4 & 5 (**C**), and the computational study of ZIP4 transmembrane (**D**). (**A**) The full-length ZIP4 has eight transmembrane (TM1 to TM8) segments crossing the plasma membrane with a large N-terminus (1–327 residues) and C-terminus facing the extracellular environment. (**B**) The N-terminus (1–327 residues) has been removed from the full-length ZIP4 at the spot indicated by figure (**A**), the membrane domain (**B**) known as the truncated ZIP4. (**C**) Sequence alignment of human ZIP proteins TMD4 and TMD5. Conserved residues involved in metal coordination/translocation are highlighted in green. The computational study of ZIP4 transmembrane top and side views (**D**). The key residues for Zn^2+^ transport are colored in red.

In humans, intracellular Zn^2+^ levels are regulated by Zinc and Iron-regulated Protein (ZIP) and Zinc Transport (ZnT) protein families^5^. ZIP transporters function to increase cytosolic Zn^2+^ levels, while the ZnT family functions to decrease cytosolic Zn^2+^ levels. Both of these protein families can be expressed in the plasma membrane or intracellular organelle membranes. The fourteen human ZIP transporters are subdivided into four subfamilies: ZIPI, ZIPII, gufA, and LIV-1^*8*^^6^. ZIP4 is one member of the largest subfamily (LIV-1) of ZIP transporters (Fig. 1A). hZIP4 is expressed on the apical surface of the intestinal wall. As such, it plays a critical role in the dietary uptake of Zn^2+^^6^. The importance of the N-terminal ExtraCellular Domain (ECD) in cellular physiology was identified when it was observed that the ECD was removed during prolonged Zn^2+^ deficiency growth conditions^7^. ZIP4 lacking the N-terminal extracellular domain (Fig. 1A) occurred in response to zinc deficiency in epithelial cells like CaCo2 and Hepa cells^8,9^, which indicates overexpression of truncated ZIP4 correlates with hypersensitivity to Zn^2+^. Therefore, the extracellular domain of ZIP4 may play an essential role in the regulatory mechanism to control Zn^2+^ transport and other transport activities^10^. Within the transmembrane (TM) domains, residues which comprise TM4 and TM5 are essential for translocation and specificity.

Mutations within the human (h) ZIP4 ECD can lead to *Acrodermatitis Enteropathica* (AE)^9^. AE is a Zn^2+^-deficiency disease that can result in disruption of the immune system and digestive homeostasis. Some of these disease-causing mutations, including AE-causing mutations can result in trafficking errors of hZIP4 to the endoplasmic reticulum^*10,11*^. In addition, hZIP4 overexpression has been found in tumor tissues, including pancreatic, liver, and brain cancer^11^. Mutations within the N-terminal ECD have been implicated in protein dysfunction^9^. In addition, AE-causing mutations near the N-terminus cleavage site prevent hZIP4 processing, leading to decreased cellular Zn^2+^ uptake^*10*^. The role of AE-causing mutations in the ECD was further explained by the crystal structure of the ECD from the black fruit bat *Pteropus Alecto* (p) ZIP4^11^. The crystal structure of pZIP4 ECD revealed a homodimer with AE mutations mapping to sites that were shown to be critical for structural stability, particularly mutations near or at a highly conserved proline-alanine-leucine (PAL) motif^11^. The PAL motif is at the dimerization interface and likely has a crucial role in protein stabilization^11^.

Bimolecular fluorescence complementation (BiFC), coimmunoprecipitation, resonance energy transfer (RET), and immunoprecipitation are commonly used to quantify the oligomer state of a protein^12^. Although co-immunoprecipitation methods provide information on protein–protein interactions, they cannot be applied in vivo to live cells because it requires the solubilization of proteins. However, fluorescence correlation spectroscopy (FCS) be used to estimate the protein–protein interactions in vivo^13^*.* FCS in particular requires a low protein expression level, making it more suitable than other methods requiring high levels of expression to studying plasma membrane transporters in their native environment^14^. Therefore, FCS can be used to monitor molecular diffusion and ligand binding for ion channels and detect the oligomer states of various receptors^15^.

**Figure 2:**
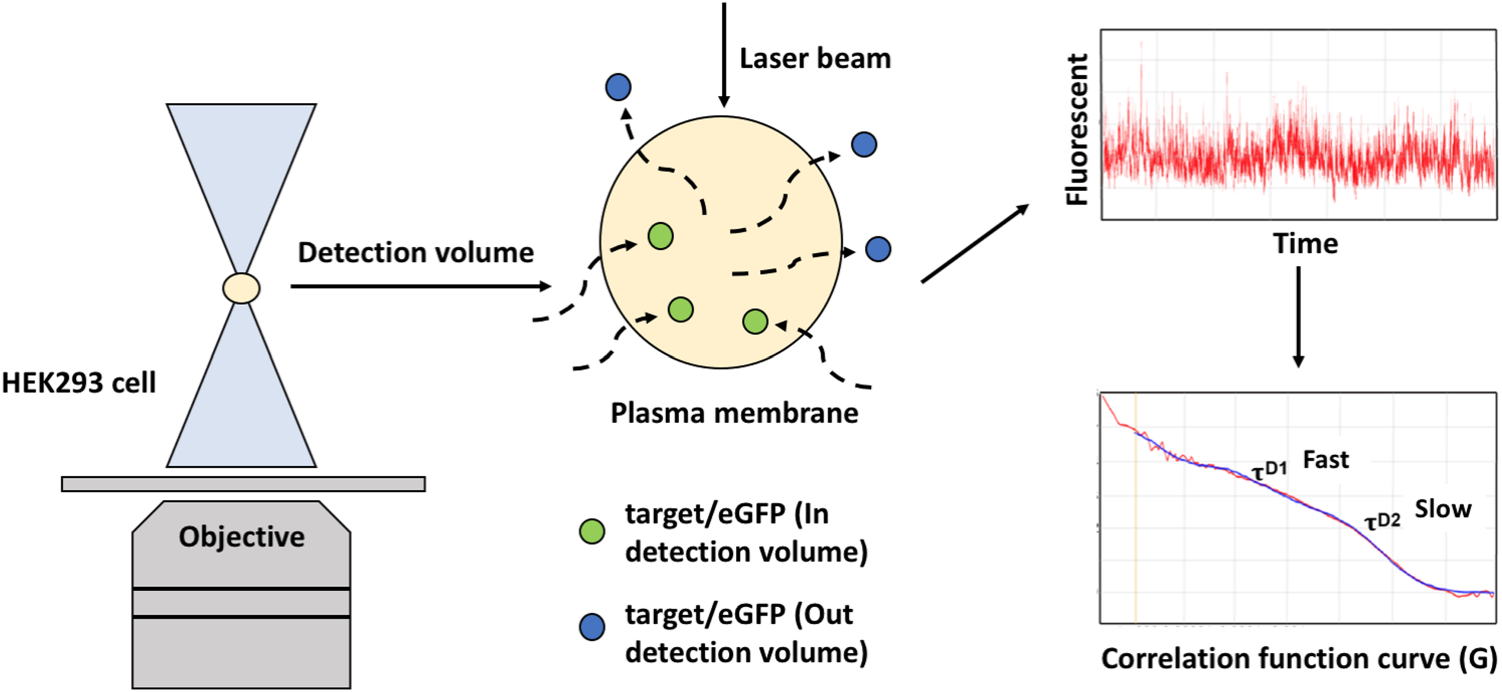
The construction of Fluorescence Correlation Spectroscopy (FCS). Combining confocal microscopy with FCS to monitor protein dynamics in living cells, the plot represents the fluctuations in the detected fluorescence intensity arising from individual fluorescent molecules (the green dots within detection volume), and it is plotted as a function of time. During imaging (center panel), eGFP diffuses in (green) and out (blue) of the detection volume in both directions. Only eGFP located within the detection volume are measured at any given time.

In an FCS measurement, the sample is illuminated by a diffraction limited area of 1 femtoliter or less. The fluorescence is generated from sample particles diffusing in and out of the illumination volume, collected by the detector, and recorded. The concept of FCS is shown in Fig. 2. The fluctuations in the detected fluorescence intensity, arising from individual fluorescent molecules that move into and out of the illumination volume, are plotted as a time function (Fig. 2)^16,17^. Combining confocal microscopy with FCS allows us to monitor protein dynamics in living cells^18–20^. The oligomer state of a protein can be determined by analyzing the amplitude of the fluctuations in fluorescence intensity measured in FCS.. Since the molecular brightness of a cluster of fluorescent molecules is directly proportional to the number of fluorescent molecules present, the molecular brightness estimates the number of fluorescent molecules within the protein complex^21^.

Recently, we revealed that the full-length hZIP4 forms a homodimer when expressed on HEK293 cell surface in vivo by FCS^13^, which is consistent with the dimeric structure of an hZIP4 computational model generated by *Rosetta* co-evolution contact data^22^. In addition, a previous study on pZIP4 ECD implicated that the ECD may play a critical role in dimeric structure formation. However, there is no consensus in the previously published literature regarding the oligomer status of truncated hZIP4 in cells (hZIP4 without ECD). In this study, we used FCS combined with confocal microscopy and determined the molecular brightness of individual fluorescence protein-tagged proteins as a measurement of their oligomer states in vivo. This study describes the analysis of the oligomer state for truncated hZIP4 functionally expressed in the plasma membrane of HEK293 cells using FCS measurement.

## Materials and methods

### Plasmids

Two hZIP4 genes were cloned into pcDNA 3.1 expression vector separately: the full-length hZIP4 gene (residues 1–647) and a truncated (without ECD) hZIP4 encoding the eight transmembrane domains (residues 328–647). The genes were cloned into a pcDNA 3.1 vector that encodes a monomeric enhanced GFP tag (eGFP) on the C-terminus of the target gene. The A206K mutation was created in each GFP to eliminate potential aggregation of the fluorescent tag^23^. CD-86/eGFP and CD-86/eGFP/eGFP plasma membrane receptors were created by fusion with C-terminus eGFP in pcDNA 3.1 vector and were used as monomer and tandem controls^24^.

### Cell culture, transfection, and reagents

Human embryonic kidney cells (HEK293T, ATCC, Cat #CRL-3216) were maintained in Dulbecco’s modified eagle’s medium (DMEM, Thermo Fisher Scientific, Invitrogen, Cat #11965092) containing 10% (v/v) fetal bovine serum (FBS, Atlanta Biologicals, Cat #S11150) and 100 units/ml penicillin and streptomycin (Life Technologies, Gibco, Cat #1935447) in a 5% CO_2_, 37 °C incubators for cell growth. For optimal results of confocal imaging and FCS measurements, HEK293 cells were plated in 35-mm poly-d-lysine coated glass-bottom culture dishes (MatTek Corporation, Cat #P35GC-1.5-14-C) at a density of 7 × 10^5^ per dish^25^ and the cells were then transfected with 50 ng pcDNA3.1 empty vector (EV) as a negative control or the same vector harboring hZIP4/eGFP or truncated hZIP4/eGFP using the Lipofectamine 3000 reagent (Thermo Fisher Scientific, Invitrogen, Cat # L3000001) according to the manufacturer’s instructions^26^. For cytosolic, monomeric, and dimeric controls, HEK293 cells were transfected with 50 ng cytosolic eGFP, CD-86/eGFP, or CD-86/eGFP/eGFP, respectively. After transfection, cells media was changed to minimal essential medium (MEM, without phenol red, Thermo Fisher Scientific, Invitrogen, Cat #2187309) for 20 h at 5% CO_2_, 37 °C^25^. Dulbecco’s phosphate-buffered saline (DPBS) was purchased from Thermo Fisher Scientific, Invitrogen, Cat #2,041,857. 0.25% Trypsin–EDTA was purchased from Thermo Fisher Scientific, Invitrogen, Cat #2085461. HEPES was purchased from Life Technologies, Gibco, Cat #1897332. Chelex-100 sodium form was purchased from Sigma, Cat #SLCC4858. Zinc sulfate monohydrate was purchased from Honeywell, Cat #307941-100G. Other metal (II) salts were purchased from Alfa Aesar.

### Confocal microscope imaging

To visualize cell surface expression of hZIP4/eGFP or truncated hZIP4/eGFP, HEK293 cells were washed with DPBS twice, then maintained in MEM with 1 mM HEPES for imaging^13^. For fluorescence confocal imaging, samples were focused with transmitted light at 10X magnification, then switched to blue light to estimate the gross localization of GFP^27^. Laser scanning confocal microscopy was performed on a Leica TCS SP5 confocal microscope. Laser beams with 488 nm wavelengths were used for eGFP excitation. The emission filter was 505 to 530 nm bandpass^28^. Single confocal sections and z-stack images were processed in ImageJ.

### Cell surface biotinylation assay

Transfected HEK293 cells were incubated with 0.5 mg/ml EZ-link Sulfo-NHS-SS-Biotin (Thermo Scientific) in cold PBS for 15 min on ice. The cells were washed twice with 200 mM glycine in cold PBS to inactivate biotin, followed by twice washing with cold PBS to remove excess biotin in the culture. The cells were then treated with lysis buffer (50 mM HEPES pH 7.4, 150 mM NaCl, 1.5 mM MgCl_2_, 1 mM EGTA, pH 8, 10% Glycerol, 1% Triton X-100 and 1X Complete Protease Inhibitor Cocktail Roche) for 1 h at 4 °C. Whole-cell lysates were centrifuged at 16,000 g at 4 °C for 15 min. 2 mg of the supernatant was incubated with 50 μl Streptavidin Sepharose High-Performance beads (GE Healthcare) for 2 h at 4 °C (the remaining supernatant was kept as input). The beads were washed four times with 1X lysis buffer before elution^29^. Then, 50 μl of 2X NuPAGE sample buffer (Invitrogen) and 10 mM DTT was added, and the beads were incubated at 75 °C for 10 min. Finally, the biotinylated proteins were separated by SDS-PAGE. Full-length hZIP4/eGFP and truncated hZIP4/eGFP were detected by Western blot using a primary antibody produced in rabbits against the C terminus of eGFP and then detected using a secondary antibody (anti-rabbit) with Horseradish Peroxidase (HRP) linked^22^.

### Transport assay by atomic absorption spectroscopy (AAS)

To analyze the function of the proteins (hZIP4/eGFP and truncated hZIP4/eGFP), HEK293 cells were transfected with either the empty vector plasmid pcDNA3.1 (50 ng) as a negative control, pcDNA3.1 with hZIP4/eGFP (50 ng), or truncated hZIP4/eGFP (50 ng)^13^. AAS was used to quantify Zn^2+^ accumulation. HEK293 cells transfected with either the empty vector plasmid pcDNA3.1 (50 ng) as a negative control, pcDNA3.1 with hZIP4/eGFP (50 ng), or truncated hZIP4/eGFP (50 ng) were measured. Twenty-four hours after transfection, cells were incubated in pre-warmed DMEM media, treated with 10% Chelex-100 v/v overnight^30^. For the time-course of Zn^2+^ accumulation, transfected HEK293 cells were incubated with 5 μM Zn^2+^ supplemented in Chelex-treated media for 0, 10, 20, 40, and 60 min at 5% CO_2_ and 37 °C. To measure the transport parameters *V*_*max*_ (the maximal velocity) and *K*_*m*_ (the concentration of divalent metal at one-half *V*_*max*_), Zn^2+^ accumulation was measured for cells incubated with varying Zn^2+^ concentrations (0, 5, 10, 15, 20, and 25 μM) supplemented in Chelex-treated media. After the incubation, all cell dishes were placed on ice and washed gently twice with ice-cold Chelex-treated media. The cells were then gently scraped from the dish and treated by acid digestion overnight before AAS measurement^31^. For competition experiments, cells were pre-incubated with 600 μM of ZnCl_2_ for 45 min at 5% CO_2_, 37 °C. After this pre-incubation step, the cells were washed and incubated with chelex-treated DMEM media. Cells were then incubated with 5 µM ZnCl_2_ and 600 µM indicated divalent transition metal and incubated for another 5 min at 5% CO_2_, 37 °C. For the controls, no additional transition metal was added^22^. AAS was employed to measure cellular Zn^2+^ levels.

### Fluorescence correlation spectroscopy (FCS) and molecular brightness analysis

For FCS measurement, cells were washed twice with 2 ml pre-warmed DPBS and then changed to 2 mL HEPES-buffered MEM (without phenol red). FCS measurements were made using a Zeiss LSM-780 confocal microscope with gallium arsenide phosphide photon detectors (Carl Zeiss, Jena, Germany). One-photon excitation with a continuous argon-ion laser was performed using a 40 × (numerical aperture 1.2) C-apochromatic water immersion objective to create an observation volume. FCS measurements were taken with the plasma membrane centered in the confocal volume above the cell nucleus of HEK293 cells transfected with the indicated eGFP-tagged protein. To center the plasma membrane in the observation volume, the photons counts per molecule in real-time were monitored while focusing upward through the plasma membrane to identify the focal plane with maximum photon counts. FCS measurements were made at room temperature (23 °C) in HEPES-buffered MEM (without phenol red) for 100 s (10 consecutive 10-s intervals). FCS data were analyzed by a digital correlator using Zeiss Zen software to calculate the autocorrelation function G(τ), which represents the time-dependent decay in fluorescence fluctuation intensity as in Eq. (1),

1 $$G\left(\tau \right)=\frac{<\delta F(t)\cdot \delta F(t+\tau )>}{{<F(t)>}^{2}}$$

where G(τ) represents the time of the change in fluorescence intensity (δF) at a one-time point (t) and at a time interval later (t + τ), then divided by the square of the average fluorescence intensity. For the autocorrelation analysis, FCS data were fit into a nonlinear least-squares two-dimensional model (for lateral diffusion time within the plasma membrane) as in Eq. (2),

2 $$\begin{aligned} G\left( \tau \right) &= 1 + AN^{ - 1} \left[ {F_{1} \left( {1 + {\raise0.7ex\hbox{$\tau $} \!\mathord{\left/ {\vphantom {\tau {\tau_{D1} }}}\right.\kern-\nulldelimiterspace} \!\lower0.7ex\hbox{${\tau_{D1} }$}}} \right)^{ - 1} + F_{2} \left( {1 + {\raise0.7ex\hbox{$\tau $} \!\mathord{\left/ {\vphantom {\tau {\tau_{D2} }}}\right.\kern-\nulldelimiterspace} \!\lower0.7ex\hbox{${\tau_{D2} }$}}} \right)^{ - 1} } \right]\\ &\left\{ {A = 1 + \left( {T_{b} e^{{ - \frac{\tau }{{\tau_{b} }}}} } \right)\left( {1 - T_{b} } \right)^{ - 1} } \right\} \end{aligned}$$

where N is the number of molecules in the observation volume, F1 with τD1 and F2 with τD2 are the fractions and diffusion times of the two components. Tb and τb represent the blinking fraction and relaxation time^32^. The fast component (on μsec scale) likely reflects the photophysical properties of the fluorophore; the slower component (on msec scale) reflects the diffusion of the protein molecule within the plasma membrane. Then, the autocorrelation curve was generated from the fluorescence intensity fluctuations as a function of particle number and diffusion time. The average dwell time of the eGFP molecules within the observation volume (τD) was calculated from the autocorrelation curve. The diffusion coefficient (D) for lateral diffusion of fluorescence-tagged proteins within the plasma membrane was calculated using Eq. (3), where ω_0_ is the radial of the observation volume^33^.

3 $$D=\frac{{\omega }_{0}^{2}}{4{\tau }_{D}}$$

The average fluorescence intensity (average photon count rate, k) for a sample was calculated by the number of fluorescent molecules (N_PSF_) and their molecular brightness (ε), as described in Eq. (4),

4 $$k={N}_{PSF}\cdot \varepsilon$$

where N_PSF_ is related to the autocorrelation function G(0), as shown in Eq.( 5),

5 $${N}_{PSF}=\frac{1}{G\left(0\right)-1}\cdot \gamma$$

where γ is the point spread function (PSF) that describes the shape of the observation volume. Overall, the molecular brightness of a dimer should be twice the brightness of a monomer. To determine the molecular brightness of monomer and dimer controls, CD-86, a monomeric plasma membrane receptor control, tagged with a single C-terminal eGFP as a monomeric control and a tandem C-terminal eGFP/eGFP as a dimer control were used^25^.

### Statistical analysis

For each experimental condition, at least three measurements (${\mathrm{x}}_{1},{\mathrm{x}}_{2},{\mathrm{x}}_{3}$) were taken. The mean and SD were calculated as following:

$$Mean:\overline{x} = \frac{{\mathop \sum \nolimits_{i = 1}^{n} x_{i} }}{n}$$

$$SD:s = \frac{{\mathop \sum \nolimits_{i = 1}^{n} (x_{i} - \overline{x})^{2} }}{n - 1}$$

P Values were calculated using the following equations:

$$\begin{gathered} H_{0} : \overline{x}_{1} = \overline{x}_{2} \;\;vs\;\;H_{a} : \overline{x}_{1} \ne \overline{x}_{2} \hfill \\ t - statistic:\;t^{*} = \frac{{ \overline{x}_{1} - \overline{x}_{2} }}{{\sqrt {\frac{{\left( {n_{1} - 1} \right)s_{1}^{2} + \left( {n_{2} - 1} \right)s_{2}^{2} }}{{n_{1} + n_{2} - 2}}\left( {\frac{1}{{n_{1} }} + \frac{1}{{n_{2} }}} \right)} }} \hfill \\ p{-}value = 2 \times P(t > \left| {t^{*} } \right|),\;\;with \, a \, degree \, of \, freedom\;\;n_{1} + n_{2} - 2. \hfill \\ \end{gathered}$$

For all experiments, gf the p-value was less than 0.05, the null hypothesis can be rejected, and it can be concluded that the two variates have significantly different means at 95% confidence level.

## Results

### Cellular localization of hZIP4 and truncated hZIP4 in HEK293 cells

**Figure 3:**
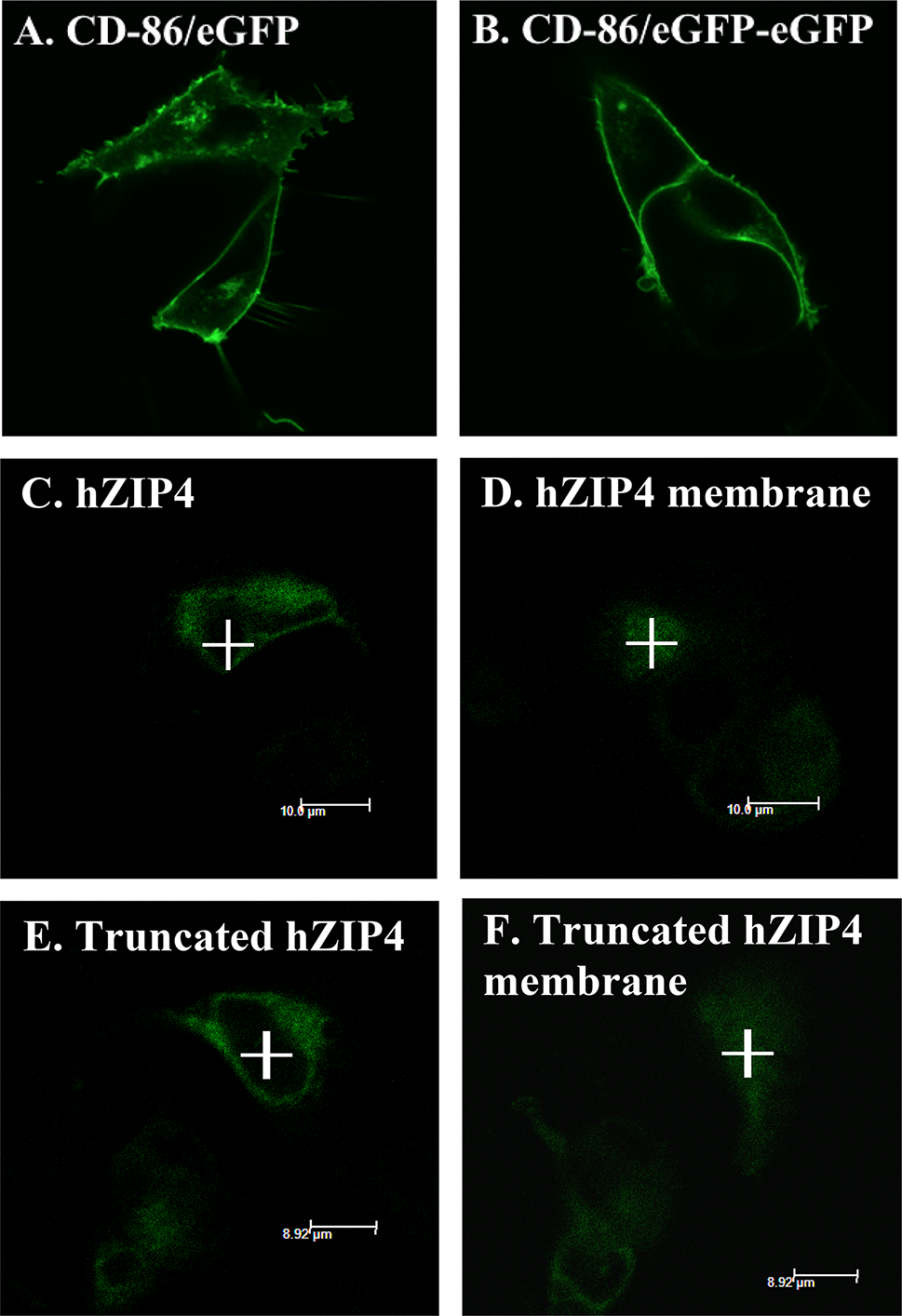
Confocal microscopy showing plasma membrane localization of hZIP4/eGFP and truncated hZIP4/eGFP. HEK293 cells were expressed of CD-86/eGFP (**A**) and CD-86/eGFP/eGFP (**B**) as monomeric and dimeric controls, respectively. (**C**) and (**D**) showed the cellular localization of full-length hZIP4/eGFP, image (**C**) was focused on the cross-section of the cell, and image (**D**) was focused on the top of the cell membrane. (**E**) and (**F**) showed the cellular localization of truncated hZIP4/eGFP, image (**E**) was focused on the cross-section of the cell, and image (**F**) was focused on the top of the cell membrane.

Confocal microscopy was used to observe the cellular localization of eGFP-tagged full-length hZIP4 and truncated hZIP4 (Fig. 3). HEK293 cells expressing CD-86/eGFP and CD-86/eGFP/eGFP were used as monomeric and dimeric controls, respectively. Monomeric CD-86/eGFP and dimeric CD-86/eGFP/eGFP were largely localized in the plasma membrane of HEK293 cells (Fig. 3A and B). Both cells transfected with hZIP4/eGFP and truncated hZIP4/eGFP (Fig. 3C–F) also exhibited fluorescence associated with the surface membrane (Fig. 3D and F), which was confirmed by surface biotinylation experiments. However, a significant amount of hZIP4/eGFP (or truncated hZIP4/eGFP) is localized intracellularly. Although there may be some contribution to the FCS analysis from hZIP4/eGFP (or truncated hZIP4/eGFP) in intracellular membranes, this contribution is unlikely to significantly alter the interpretation of the FCS analysis as regardless of where the proteins are localized, the oligomeric state will remain the same.

### The impact of hZIP4 extracellular domain on zinc transport

**Figure 4:**
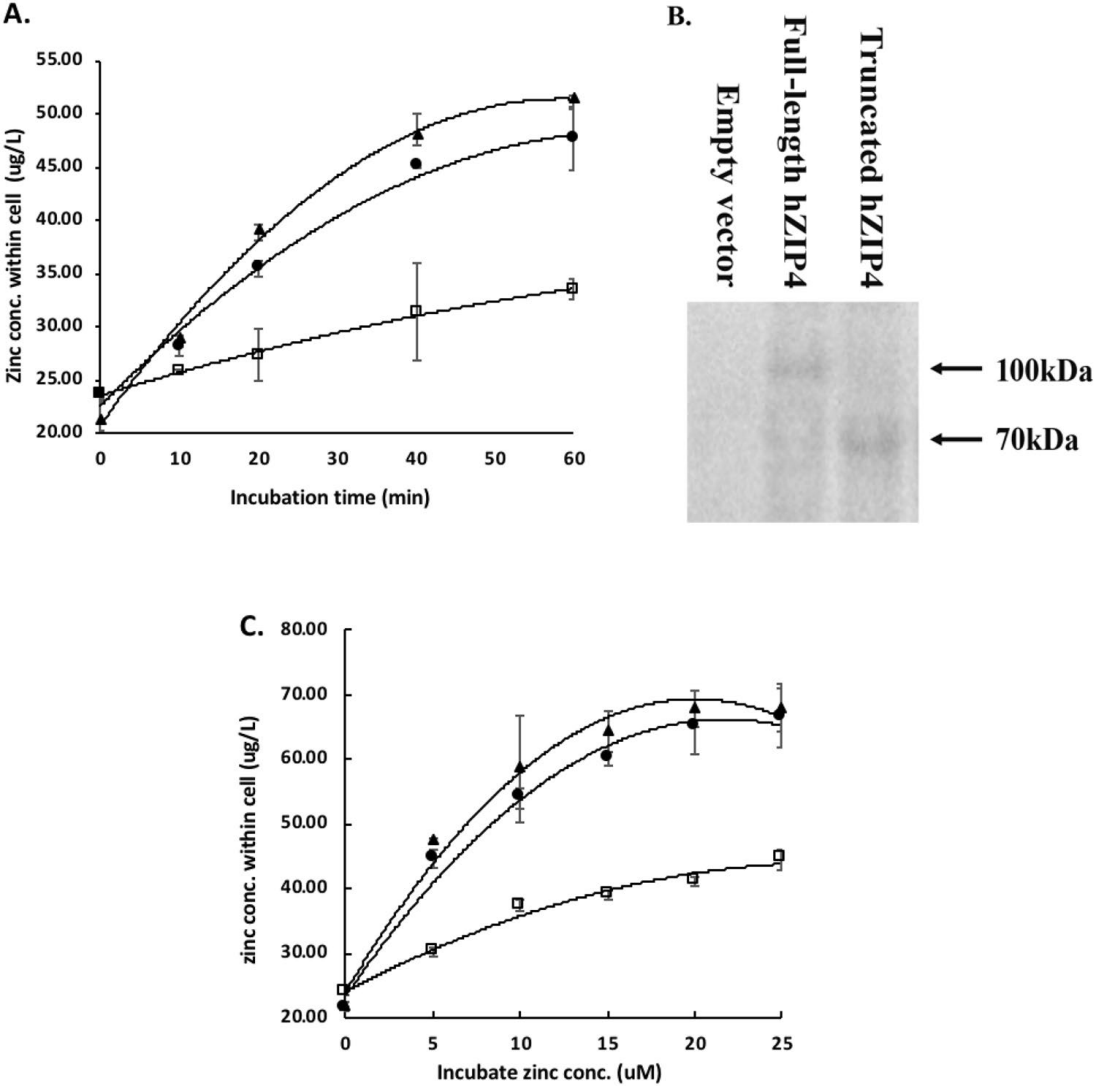
Kinetics of hZIP4 and truncated hZIP4 zinc transport. (**A**) Zn^2+^ uptake in HEK293 cells expressed full-length hZIP4 (▲), truncated hZIP4 (●), or empty vector (□) as a function of time. (**B**) full-length hZIP4 and truncated hZIP4 expression level on the plasma membrane of HEK293 determined by biotinylation assay. Full-length imaging is shown in Supplement Fig. S1. (**C**) Zn^2+^ uptake in HEK293 cells expressed full-length hZIP4 (▲), truncated hZIP4 (●), or empty vector (□) at various Zn^2+^ concentrations, and the transport parameters, *K*_*m*_ and *V*_*max*_ of full-length hZIP4 were calculated as 3.14 ± 0.2 µM and 0.24 ± 0.01, respectively. *K*_*m*_ and *V*_*max*_ of truncated hZIP4 are 3.46 ± 0.3 µM and 0.23 ± 0.01, respectively.

The surface expression of full-length and truncated hZIP4 was further confirmed by performing a biotinylation experiment of intact, transfected HEK293 cell.. After lysis, biotinylated proteins were identified after electrophoresis under reducing conditions. In the biotinylated membrane fraction, the bands at around 100 kDa and around 70 kDa were observed as full-length hZIP4/eGFP and truncated hZIP4/eGFP, respectively (Fig. 4B). It is important to note here that as our FCS experiments require low levels of protein expression, consequently these bands are weak. No significant difference between the density of the bands indicated that the expression levels of full-hZIP4/eGFP and truncated hZIP4/eGFP were similar. The relatively low sensitivity of the bands is likely due to the low expression level of those proteins on the plasma membrane. Nevertheless, the surface expression of the N-terminal truncated hZIP4/eGFP was comparable to that of the full-length protein.

Zn^2+^ transport kinetics were determined for full-length hZIP4 and truncated hZIP4 in HEK293 cells^31^. There was a significant increase in Zn^2+^ accumulation for HEK293 cells expressed full-length hZIP4 and truncated hZIP4 compared with cells transfected with the empty vector control (Fig. 4A). The *K*_*m*_ values for full-length and truncated hZIP4 were calculated to be 3.14 ± 0.2 µM and 3.46 ± 0.3 µM (Fig. 4C), respectively. Finally, *V*_*max*_ values between the full-length hZIP4 (0.24 ± 0.01) and truncated hZIP4 (0.23 ± 0.01) were indistinguishable. This *V*_*max*_ data, combined with Western blot detection of cell surface biotinylation, indicates that the maximal velocity of Zn^2+^ transport is indistinguishable between the full-length and truncated transporter.

**Figure 5:**
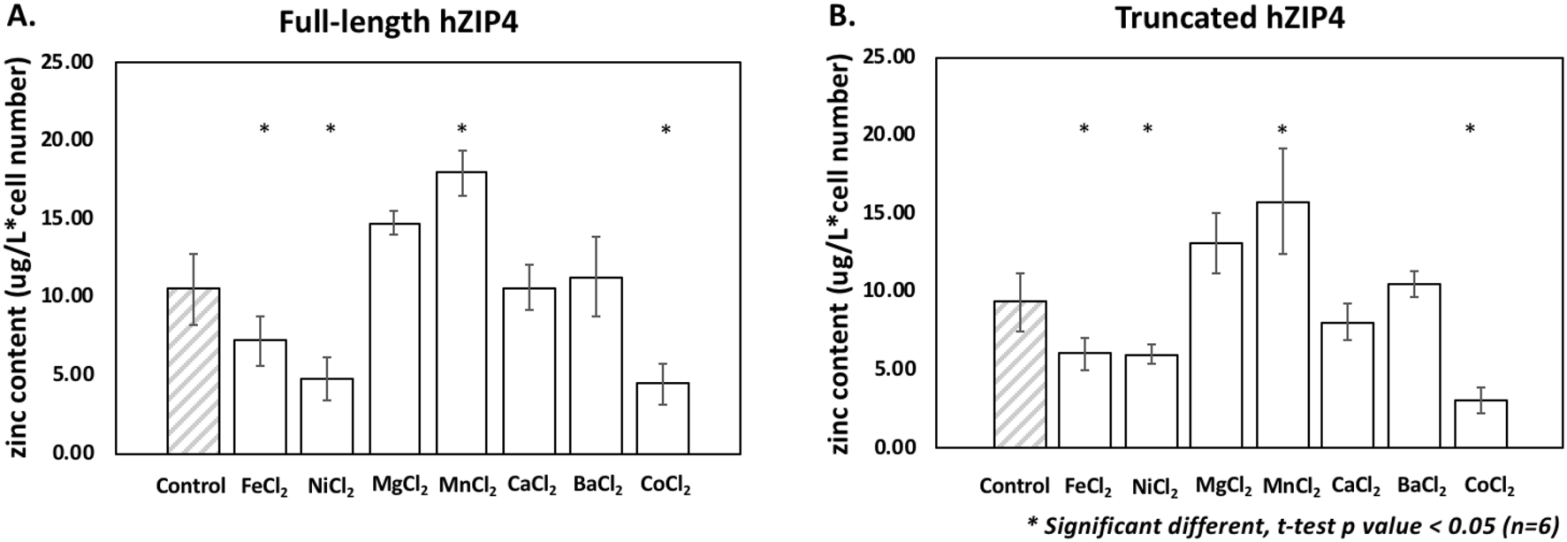
Competition of zinc transport with a series of divalent cations. To exam which divalent cations inhibit hZIP4-meidated Zn^2+^ transport. HEK293 cells expressing hZIP4 (**A**) or truncated hZIP4 (**B**) were pre-incubated in 600 µM ZnCl_2_ (control), FeCl_2_, NiCl_2_, MgCl_2_, MnCl_2_, CaCl_2_, BaCl_2_ or CoCl_2_ complemented media, separately. The transport was initiated by adding 5 µM Zn^2+^. * Statistically significant, t-test* p* value < 0.05 (n = 3).

We next explored the impact of the extracellular domain on hZIP4 substrate specificity via screening the divalent cations that inhibit hZIP4-mediated zinc uptake. HEK293 cells expressed full-length hZIP4 and truncated hZIP4 were preincubated in cold uptake assay buffer containing 600 µM ZnCl_2_ (control), FeCl_2_, NiCl_2_, MgCl_2_, MnCl_2_, CaCl_2_, BaCl_2_ or CoCl_2_, separately. The uptake assay was initiated by adding 5 µM ZnCl_2_. For both full-length hZIP4 transporter and truncated hZIP4, Zn^2+^ transport was significantly inhibited by Fe^2+^, Ni^2+^, and Co^2+^ (Fig. 5A, B).

### The effects of hZIP4 extracellular domain on hZIP4 oligomeric state formation

FCS analysis was used to determine the oligomerization of full-length hZIP4/eGFP and truncated hZIP4/eGFP. FCS was measured on the upper plasma membrane of transfected HEK293 cells. hZIP4/eGFP (and truncated hZIP4/eGFP) appeared to be expressed on the cell surface by confocal microscopy (Fig. 3D and F), consistent with the cell surface biotinylation results and functional zinc transport assays described earlier.

**Figure 6:**
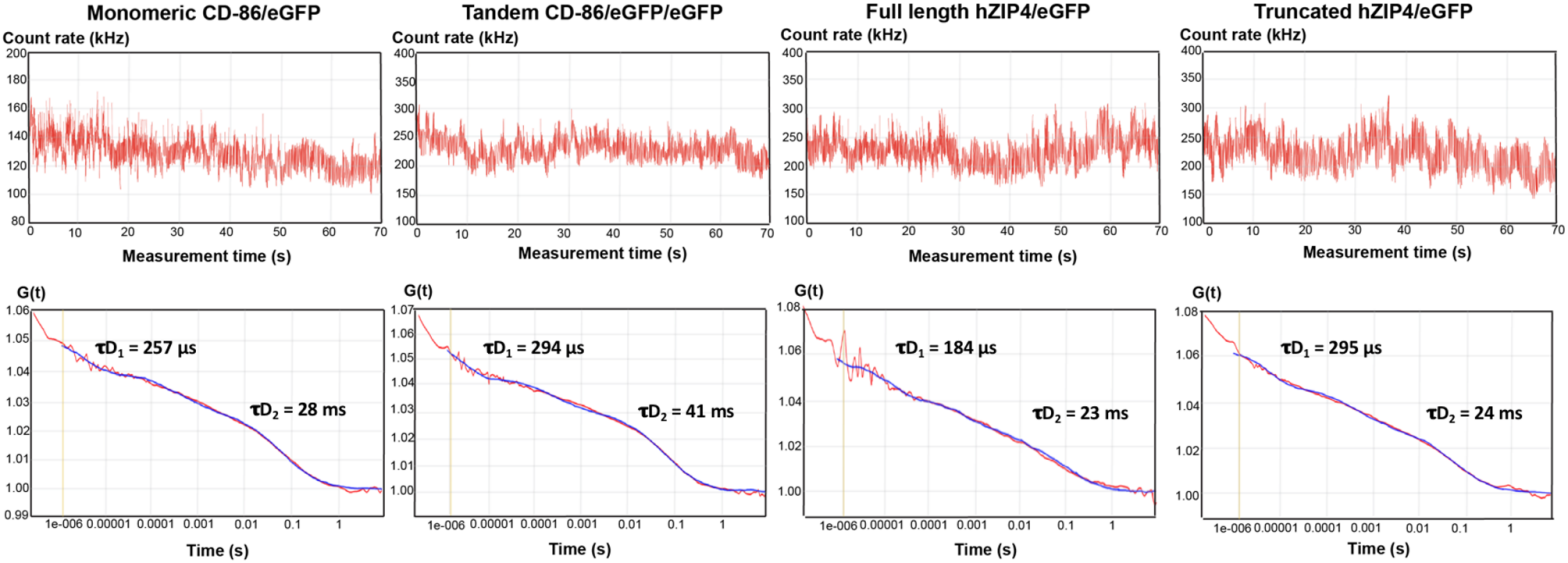
Fluorescence correlation spectroscopy (FCS) autocorrelation curves of eGFP-tagged membrane proteins. FCS recordings were made on the plasma membrane of transiently transfected HEK293 cells expressing the indicated eGFP-tagged proteins. CD-86/eGFP and CD-86/eGFP/eGFP are the monomeric and dimeric controls, respectively. Fluorescence intensity fluctuations from recording outside the range of 50–250 kHz were excluded. Autocorrelation curve of FCS data (red line) with a model curve fitting a two-dimensional two-component model.

For FCS experiments, fluorescence intensity fluctuations generated by movement of eGFP-tagged hZIP4 (and eGFP-tagged truncated ZIP4) into and out of a diffraction-limited spot were collected, as shown in Fig. 6. The fluorescence intensity fluctuation was fit to the autocorrelation function G(τ) using a non-linear least-squares model (Fig. 6) as described in methods. The average dwell time (τD) of the eGFP-tagged hZIP4 (and eGFP-tagged truncated ZIP4) transporter within the observation volume represents its diffusion time within the plasma membrane, and it is calculated from the mid-point of the autocorrelation curve. The autocorrelation curves shown in Fig. 6 were fitted by a two-component model with a very fast diffusion time (τD_1_) that reflects the photophysical characteristic of the eGFP tag while a slow diffusion time (τD_2_) reflecting the translational movement of the hZIP4/eGFP (and the truncated hZIP4/eGFP) within the cell plasma membrane.

Fluorescent fluctuation intensity in Fig. 6 reveals similar average photon count rates for the full-length hZIP4/eGFP and truncated hZIP4/eGFP and the dimeric CD-86/eGFP/eGFP control. In Fig. 6, the amplitude of the autocorrelation curve is inversely related to the number of fluorescent proteins appeared in the observation volume. The molecular brightness values of the full-length hZIP4/eGFP and truncated hZIP4/eGFP were calculated using the number of fluorescent molecules calculated from the amplitude of the autocorrelation curves and the average photon count that recorded from the fluorescence fluctuation intensity signal in Fig. 6. An estimated molecular brightness of each sample was calculated as counts per second per molecule (CPSM) showed in Table 1. The molecular brightness of the full-length hZIP4/eGFP and truncated hZIP4/eGFP are close to the molecular brightness of the dimeric control.

**Table 1:** Brightness comparison for FCS measurements**.**

Sample	Brightness (CPSM)	Reduced X^2^	*n*
Cytosolic eGFP	4500 ± 700	(14 ± 7) × 10^–7^	7
Monomeric CD-86/eGFP	4600 ± 900	(24 ± 9) × 10^–7^	6
Tandem CD-86/eGFP/eGFP	8300 ± 900	(20 ± 9) × 10^–7^	6
Full-length hZIP4/eGFP	8100 ± 1800	(16 ± 7) × 10^–7^	6
Truncated hZIP4/eGFP	8200 ± 2000	(10 ± 5) × 10^–7^	6

Calculated brightness of molecules tagged with eGFP for HEK293 cells expressed cytosolic, membrane-bound monomeric eGFP control, membrane-bound dimeric eGFP control, full-length hZIP4/eGFP, and truncated hZIP4/eGFP protein, respectively. Brightness values are reported as counts per second per molecule (CPSM). X^2^ refers to the goodness of fit of the data to the two-component model. Data represent the mean  ±  the standard error of the mean for the number of cells examined (n).

## Discussion

hZIP4 encodes eight-transmembrane domains and a large N-terminus extracellular domain (ECD with 327 residues)^*8*^. The ECD is cleaved from the transporter when cells are Zn^2+^ deficient. Although the highly conserved PAL motif within the ECD may play a crucial role in protein stabilization, the data presented here supports the conclusion that the TM segments are sufficient to form dimers when expressed on the plasma membrane in vivo. In addition, there is no significant impact on Zn^2+^ transport properties within the cells expressing hZIP4 without the ECD. This is relevanet as the large ECD has been shown to be cleaved from the holo-transporter when cells are Zn^2+^-deficient. This proteolysis, leaving only the transmembrane domains of ZIP4 does not impact transition metal inhibition studies, and the functional effect of this post-translational modification remains unresolved.

An interesting outcome from inspection of the data shown here is that high levels of Mn^2+^ increases Zn^2+^ uptake in our competition experiments. This change in Zn^2+^ transport could be a consequence of Mn^2+^ induced Zn^2+^ transport. Related to this observation, it has previously been shown that ZIP8 and ZIP14 are broad spectrum transition metal transporters that can translocatd Mn^2+^ across membranes. More recently, it has been suggested that within a conserved sequence of TM5 residues, a change from His to Glu defines the manganese transport properties of ZIP8 and ZIP14 when compared to ZIP4 and other ZIP transporters (Fig. 1)^34^. However, this manuscript also noted that this postulation has not been experimentally confirmed. Based on our inhibition studies, we propose that this sequence difference does not preclude Mn^2+^ from coordinating within the transmemebrane domains, along with Zn^2+^ features outside of this singular residue change contribute to Mn^2+^ transport within the ZIP family of proteins.

## Supplementary Information

Supplementary Information.

## Data Availability

The datasets used and/or analyzed during the current study are available from the corresponding author upon reasonable request.
