# Supplementary material for: The transmembrane domains mediate oligomerization of the human ZIP4 transporter in vivo
Source: Sci Rep. 2022 Dec 6;12:21083. doi: 10.1038/s41598-022-24782-6 (PMC9727157; doi:10.1038/s41598-022-24782-6)
Supplement: Supplementary file 1 — Supplementary Information. [file 41598_2022_24782_MOESM1_ESM.docx]

Supplemental Data

**The transmembrane domains mediate oligomerization of the human ZIP4 transporter in vivo.**

**Yuting Liu^1^, Elizabeth M. Bafaro^1^, Ann Cowan^2^, Robert E. Dempski^1,^**^*^

^1^Department of Chemistry and Biochemistry, Worcester Polytechnic Institute, Worcester, MA 01609, U.S.

^2^Department of Molecular Biology and Biophysics; Center for Cell Analysis and Modeling, University of Connecticut, Farmington, CT 06030, U.S.

^*^To whom correspondence should be addressed: Robert E. Dempski, Department of Chemistry and Biochemistry, Worcester Polytechnic Institute, 100 Institute Road, Worcester, MA 01609, USA, Tel: (508) 831-4193; E-mail: rdempski@wpi.edu


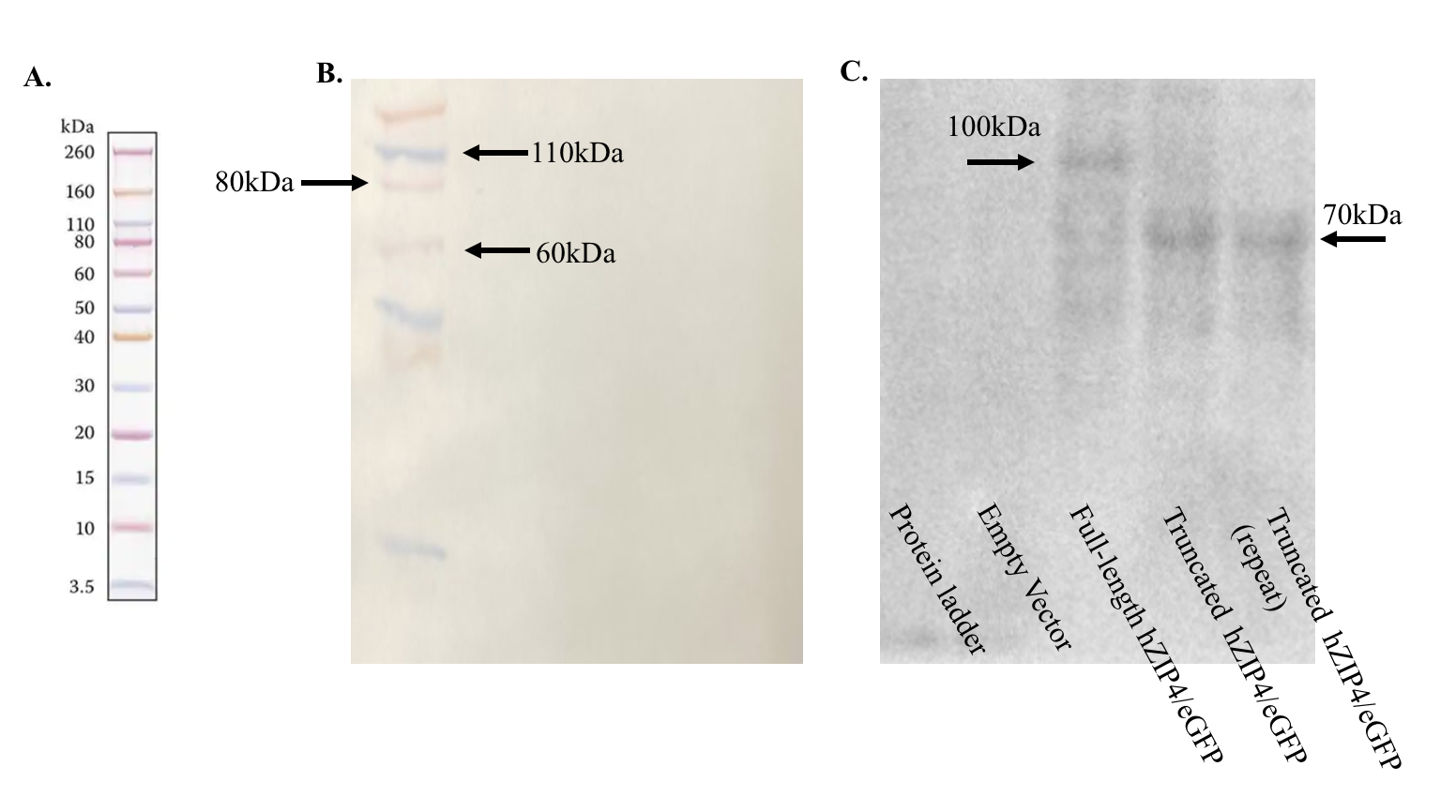


**Supplement Figure 1.** **(A)** Invitrogen™ Novex™ Sharp Pre-stained Protein Standard (Cat. LC5800). **(B)** The full-length nitrocellulose membrane for gel transfer. Novex™ pre-stained protein standard marker is shown in the first column. **(C)** Imaging of the full-length nitrocellulose membrane **(B)** after the exposure. The first column is the protein standard ladder, followed by the empty vector, the full-length hZIP4, and the truncated hZIP4. Band size was estimated by the corresponding protein ladder marker in the original nitrocellulose membrane.
